# Assessing the quality and value of metabolic chart data for capturing core outcomes for pediatric medium-chain acyl-CoA dehydrogenase (MCAD) deficiency

**DOI:** 10.1186/s12887-023-04393-4

**Published:** 2024-01-13

**Authors:** Ryan Iverson, Monica Taljaard, Michael T. Geraghty, Michael Pugliese, Kylie Tingley, Doug Coyle, Jonathan B. Kronick, Kumanan Wilson, Valerie Austin, Catherine Brunel-Guitton, Daniela Buhas, Nancy J. Butcher, Alicia K. J. Chan, Sarah Dyack, Sharan Goobie, Cheryl R. Greenberg, Shailly Jain-Ghai, Michal Inbar-Feigenberg, Natalya Karp, Mariya Kozenko, Erica Langley, Matthew Lines, Julian Little, Jennifer MacKenzie, Bruno Maranda, Saadet Mercimek-Andrews, Aizeddin Mhanni, John J. Mitchell, Laura Nagy, Martin Offringa, Amy Pender, Murray Potter, Chitra Prasad, Suzanne Ratko, Ramona Salvarinova, Andreas Schulze, Komudi Siriwardena, Neal Sondheimer, Rebecca Sparkes, Sylvia Stockler-Ipsiroglu, Kendra Tapscott, Yannis Trakadis, Lesley Turner, Clara Van Karnebeek, Anthony Vandersteen, Jagdeep S. Walia, Brenda J. Wilson, Andrea C. Yu, Beth K. Potter, Pranesh Chakraborty

**Affiliations:** 1 School of Epidemiology and Public Health, University of Ottawa, Ottawa, Canada; 2 Clinical Epidemiology Program, Ottawa Hospital Research Institute, Ottawa, Canada; 3 Department of Pediatrics, Children’s Hospital of Eastern Ontario and University of Ottawa, 401 Smyth Road, Ottawa, ON K1H 8L1 Canada; 4 The Hospital for Sick Children/University of Toronto, Toronto, Canada; 5 Bruyère Research Institute, Ottawa, Canada; 6 Department of Medicine, University of Ottawa, Ottawa, Canada; 7 Le Centre Hospitalier Universitaire Ste-Justine, Montreal, Canada; 8 McGill University Health Centre, Montreal, Canada; 9 The Hospital for Sick Children Research Institute/University of Toronto, Toronto, Canada; 10 Department of Medical Genetics, University of Alberta/Stollery Children’s Hospital, Edmonton, Canada; 11 IWK Health Centre/Dalhousie University, Halifax, Canada; 12 Health Sciences Centre Winnipeg/University of Manitoba, Winnipeg, Canada; 13 London Health Sciences Centre/Western University, London, Canada; 14 McMaster Children’s Hospital, Hamilton, Canada; 15 Hamilton Health Sciences Centre/McMaster University, Hamilton, Canada; 16 Janeway Children’s Hospital/Memorial University, St John’s, Canada; 17 CIUSSSE-CHUS, Université de Sherbrooke, Sherbrooke, Canada, Sherbrooke, Canada; 18 BC Children’s Hospital/University of British Columbia, Vancouver, Canada; 19 Alberta Children’s Hospital/University of Calgary, Calgary, Canada; 20 Emma Center for Personalized Medicine, Amsterdam University Medical Centers, Amsterdam, The Netherlands; 21 Kingston Health Sciences/Queen’s University, Kingston, Canada; 22 Newborn Screening Ontario, Ottawa, Canada

**Keywords:** MCAD deficiency, Core outcome set, Data quality

## Abstract

**Background:**

Generating rigorous evidence to inform care for rare diseases requires reliable, sustainable, and longitudinal measurement of priority outcomes. Having developed a core outcome set for pediatric medium-chain acyl-CoA dehydrogenase (MCAD) deficiency, we aimed to assess the feasibility of prospective measurement of these core outcomes during routine metabolic clinic visits.

**Methods:**

We used existing cohort data abstracted from charts of 124 children diagnosed with MCAD deficiency who participated in a Canadian study which collected data from birth to a maximum of 11 years of age to investigate the frequency of clinic visits and quality of metabolic chart data for selected outcomes. We recorded all opportunities to collect outcomes from the medical chart as a function of visit rate to the metabolic clinic, by treatment centre and by child age. We applied a data quality framework to evaluate data based on completeness, conformance, and plausibility for four core MCAD outcomes: emergency department use, fasting time, metabolic decompensation, and death.

**Results:**

The frequency of metabolic clinic visits decreased with increasing age, from a rate of 2.8 visits per child per year (95% confidence interval, 2.3–3.3) among infants 2 to 6 months, to 1.0 visit per child per year (95% confidence interval, 0.9–1.2) among those ≥ 5 years of age. Rates of emergency department visits followed anticipated trends by child age. Supplemental findings suggested that some emergency visits occur outside of the metabolic care treatment centre but are not captured. Recommended fasting times were updated relatively infrequently in patients’ metabolic charts. Episodes of metabolic decompensation were identifiable but required an operational definition based on acute manifestations most commonly recorded in the metabolic chart. Deaths occurred rarely in these patients and quality of mortality data was not evaluated.

**Conclusions:**

Opportunities to record core outcomes at the metabolic clinic occur at least annually for children with MCAD deficiency. Methods to comprehensively capture emergency care received at outside institutions are needed. To reduce substantial heterogeneous recording of core outcome across treatment centres, improved documentation standards are required for recording of recommended fasting times and a consensus definition for metabolic decompensations needs to be developed and implemented.

**Supplementary Information:**

The online version contains supplementary material available at 10.1186/s12887-023-04393-4.

## Background

The mitochondrial fatty acid oxidation disorder, medium-chain acyl-CoA dehydrogenase (MCAD) deficiency, is one of the most common inherited metabolic diseases, with an estimated birth prevalence as high as 1 in 12,000 in Canada [1, 2]. The MCAD enzyme is involved in the breakdown of medium-chain fatty acids, [3] which is required for sustaining euglycemia after the depletion of glycogen stores, for example, during high energy activities, when fasting, or when unwell with fever or vomiting [4, 5]. Deficiency of this enzyme markedly increases the risk of life-threatening manifestations during such periods of catabolic stress, including metabolic decompensations characterized by hypoketotic hypoglycemia, lethargy, and/or seizures [6, 7]. Treatment typically involves the avoidance of prolonged fasting, medical monitoring during periods of illness, and the provision of rapidly available carbohydrates [8]. Longer-term preventive interventions, such as carnitine supplementation, are used in some children with MCAD deficiency, although evidence regarding their benefits and harms is lacking [9–11]. Newborn screening has transformed outcomes for children with MCAD Deficiency by allowing early diagnosis and presymptomatic treatment to prevent mortality (1, 2, 30, 31). Newborn screening panels in Canada vary from province to province; most provinces began screening for MCAD deficiency in the early 2000s (range 2001–2012).

To improve care and long-term outcomes for children with MCAD deficiency, rigorous approaches to evaluation of treatments are needed, informed by reliable, sustainable, and longitudinal measurement of clinically meaningful and patient-centred outcomes [12, 13]. A core outcome set (COS) is a small group of priority outcomes agreed upon by stakeholders interested in a specific health condition with the goal of encouraging the standardized measurement and reporting of endpoints measured during clinical trials for that condition [14, 15]. The development and implementation of COSs can support the synthesis of evidence and the comparison of findings across clinical trials where appropriate. These outcomes can also be collected as part of a high-quality disease registry to establish robust observational data over time and to facilitate registry-based randomized trials, where a trial is implemented in a registry platform that incorporates rigorous outcome measurement [16]. There is a particular need for multi-centre and international collaboration in rare disease settings, given the small number of patients in any single centre. A COS can facilitate such collaboration in rare disease research as part of the harmonization of data on long-term outcomes and treatment effectiveness in small populations, thereby increasing the robustness of data pooling and thus improving the quality of evidence.

We recently developed a COS for children with MCAD deficiency as part of the Core Outcome Measures in Effectiveness Trials (COMET) initiative (www.comet-initiative.org), [17] relying on: (i) a systematic review of prior studies of MCAD deficiency to derive a potential list of relevant outcomes; [18] and (ii) a multi-stakeholder consensus approach (Delphi survey and workshop) involving patients and families, clinicians, and policymakers [19]. The final COS comprised eight core outcomes for children up to age 12 years diagnosed with MCAD deficiency, four of which could be ascertained from a child’s metabolic chart and therefore were identified as being of primary interest for the present study: emergency department use, fasting times, metabolic decompensation, and death [19]. Some outcomes, notably emergency department use, may alternatively be measured using population-wide healthcare administrative data, as demonstrated in a previous Ontario-based study from our group [20]. However, these administrative records often lack the detailed clinical information needed to reliably measure outcomes such as fasting and episodes of metabolic decompensation.

To facilitate prospective collection of these clinical outcomes and thereby support observational registries and clinical trials for children with MCAD deficiency, there is a need to establish the feasibility and sustainability of measuring these outcomes in routine clinical settings, and for outcomes other than death, the opportunity for ascertainment on a long-term and regular basis [21]. To assess the quality of existing metabolic chart core outcomes data and their future suitability for prospective measurement during metabolic clinic visits, we used existing cohort data to investigate the frequency of clinic visits and quality of metabolic chart data for selected outcomes.

## Materials and methods

### Data source and eligibility

The Canadian Inherited Metabolic Diseases Research Network (CIMDRN) established a consent-based cohort of nearly 800 children across Canada diagnosed with one of 31 Inherited Metabolic Diseases (IMD), including MCAD deficiency, [22] and included collection of clinical data from the metabolic charts for enrolled children, from birth up to a maximum of 11 years of age. The cohort was developed as a platform to support research that broadly seeks to understand health care and outcomes in this pediatric population.

Children were eligible for the CIMDRN cohort if they were born between January 1, 2006 and December 31, 2015, and received care for a confirmed diagnosis of MCAD deficiency at one of the 13 participating treatment centres between birth and March 31, 2017. Research staff at participating centres retrospectively abstracted data from electronic and/or paper charts depending on the type of metabolic chart in use at the participating centre at the time of each visit. At baseline, abstracted data pertained to participant and family characteristics, medical history, source of ascertainment, and diagnostic tests completed. For each visit to the metabolic clinic after diagnosis, the results of follow-up tests, disease-specific outcomes, treatment, and acute and chronic diagnoses were abstracted. Data fields were selected to support anticipated research questions related to health care and outcomes and to capture information likely to be present in existing metabolic charts. All data were entered as open- and closed-ended responses in a series of study-specific, web-based data collection forms developed with extensive input from metabolic clinicians across Canada who were members of CIMDRN. The data collection forms were submitted to and stored on a central study database in Research Electronic Data Capture (REDCap), [23, 24] a secure, web-based software platform hosted at the Children’s Hospital of Eastern Ontario Research Institute; these forms can be obtained by contacting the corresponding author). In addition to the careful design of intuitive data collection tools and regular communication with treatment centre research staff, in order to maintain data quality, the data were subject to a detailed verification process by staff at the CIMDRN central office, including a review of each participant’s full dataset and periodic monitoring of summary measures [22]. A unique, study-specific patient identifier was assigned to each participant in lieu of names and other identifying information to uphold patient confidentiality. Ethics approval for the protocol outlining cohort enrollment, clinical data collection, and analysis was granted by the Children’s Hospital of Eastern Ontario Research Ethics Board, the Ottawa Health Science Network Research Ethics Board, and the research ethics board at each participating centre.

For the present study, we conducted an analysis of these previously abstracted metabolic chart data from enrolled children with MCAD deficiency. Children were excluded if they had no recorded clinic visits after initial enrollment, for example, due to complete absence of data entry or death prior to their initial clinic visit. Children were followed until the study end date of March 31, 2017 unless they were deceased or discharged from a participating metabolic centre during the study period (e.g., due to relocation to a centre not participating in the cohort study). Children who moved to a participating centre from a non-participating centre during the study period were followed from the date of their first recorded clinic visit with the participating centre.

For each participant, data were abstracted from charts and entered into REDCap chronologically, starting from birth or the youngest age of a first recorded clinic visit at a participating centre. If data entry for a participant ended before March 31, 2017 and we were unable to confirm a death or discharge from the clinic, we used all available data for that participant in the analysis. An exception was when calculating visit frequencies (rates per child per year) that involved summing follow-up time; for these analyses, we considered children with incomplete data to be lost to follow-up at the end of the oldest age group to which they were known to be followed for the complete period.

### Analysis

We described the demographic characteristics (e.g., year of birth, sex, consenting treatment centre) and baseline clinical characteristics (e.g., ascertainment method and neonatal complications) using frequencies and percentages. We calculated confidence intervals for incidence rates using the exact Poisson distribution or the normal approximation to the Poisson distribution as appropriate. Confidence intervals for means were expressed using the standard normal distribution. All cell counts representing fewer than five children were suppressed as “ < 5” to reduce the risk of identifying participants, in accordance with research ethics requirements. SAS software® version 9.4 (SAS Institute Inc., Cary, North Carolina, USA) was used for all statistical analyses.

Data collection intervals among participants varied, depending on each child’s schedule of visits to the metabolic clinic. We considered the frequency of visits as an indicator of the frequency of opportunity for outcome measurement. To determine the potential future feasibility of collecting core outcomes prospectively by relying on existing clinical encounters, we reported the frequency of visits to the metabolic clinic and telehealth encounters over time, expressed as rates per child per year, calculated as the total number of visits divided by total person-time of follow-up. Visit rates are presented by child age using 6-month intervals in the first year of life and 2-year intervals thereafter. They exclude visits occurring prior to two months of age in order to focus on visits occurring after a complete diagnosis is typically established, and when core outcome measurement may be most relevant. We explored variation in the frequency of clinic visits among participating centres treating five or more children by presenting results separately for each centre.

To evaluate the quality of data for each of the outcomes of interest, we explored the four core outcomes of interest and their components, guided by Kahn et al.’s framework [25] covering three data quality concepts: completeness, conformance, and plausibility. To address completeness, we examined the extent to which individual components of a particular outcome were captured in abstracted data. To address conformance, we measured the extent to which data were entered in the proposed format and whether there existed variation in how outcomes were measured, abstracted, or recorded across sites. To address plausibility, we examined whether aggregate measures reflected a reasonable measurement or health trajectory over time for a child with MCAD deficiency, expressed as rates or summary measures of core outcomes across age groups as appropriate. For one of the core outcomes of interest, metabolic decompensation, there exists no widely accepted clinical definition. Thus, to ascertain whether metabolic chart-abstracted data may be used to measure this outcome for children with MCAD deficiency, we first identified acute clinical manifestations commonly associated with metabolic decompensation (e.g., hypoglycemia, seizures). Next, two pediatric metabolic physicians (PC, MTG) made independent inferences about whether each event identified by abstracted data constituted a true metabolic decompensation, judging each event as a yes (decompensation) or no (not a decompensation). The physicians relied solely on the abstracted data items to make this judgement. We measured agreement between the two raters using Cohen’s kappa coefficient.

## Results

### Sample characteristics

There were 132 children with a confirmed diagnosis of MCAD deficiency enrolled in the CIMDRN cohort. Eight children were excluded from further analyses since they were either deceased within the neonatal period, lost-to-follow-up prior to their first clinic visit, or data abstraction from their metabolic chart had not been initiated at the time of analysis. Children were distributed across the eligible years of birth, with the highest proportion born in 2014–2015 (Table 1). Slightly fewer than half (44%) of children were female. The proportions of children recruited from different metabolic centres generally reflected the population catchment associated with those centres. Children were enrolled from metabolic centres located in seven provinces across Canada. Ascertainment was almost exclusively achieved through newborn screening, occasionally in combination with other methods such as family history/cascade testing or symptomatic presentation. Amongst the 104 infants for whom the presence or absence of neonatal complications were available, hypoglycemia (isolated or with other neonatal complications) was reported in 13%, and is notable as a complication possibly associated with MCAD deficiency. An additional 18% of infants did not have documented neonatal hypoglycemia but were noted to have other neonatal complications. These included requirement for intravenous fluids (7%), respiratory distress (7%), need for antibiotics (7%), and jaundice (6%). The median follow-up time was 5.2 years (Interquartile Range [IQR] = 3.0–8.4 years) among participating children, with 113 children (91%) followed from birth until the end of the data collection period. Eleven children had less than complete follow-up during the study period due to late enrollment (e.g., moving from a non-participating centre) or were lost to follow-up prior to the completion of the study (e.g., discharge, incomplete data entry).

**Table 1:** Participant characteristics

Characteristic	Frequency (%)
**Year of birth** (*n* = 124)	
2006–2007	20 (16%)
2008–2009	27 (22%)
2010–2011	18 (15%)
2012–2013	28 (23%)
2014–2015	31 (25%)
**Sex (female)** (*n* = 124)	54 (44%)
**Consenting treatment centre** (*n* = 124)	
Centre A	30 (24%)
Centre B	29 (23%)
Centre C	15 (12%)
Centre D	10 (8%)
Centre E	10 (8%)
Centre F	8 (7%)
Centre G	5 (4%)
Centre H	5 (4%)
Other participating centres^a^	12 (10%)
**Ascertainment** (*n* = 124)^b^	
Newborn screening method only	107 (86%)
Newborn screening and other method(s)	13–16
Other method(s) only	< 5
**Neonatal complications (yes)** (*n* = 104)	32 (31%)
Hypoglycemia	13 (13%)
Other complications without documented hypoglycemia (e.g. respiratory distress, antibiotics, IV fluids, and/or jaundice)	19 (18%)

^a^ “Other participating centres” are those who enrolled fewer than five participants; the total number of participants from these centres is presented in the table

^b^Based on CIMDRN’s privacy policy, cells representing less than five children are suppressed as “ < 5” and a range is provided in the preceding cell to ensure the small cell size is not calculable

### Visits to the metabolic clinic

Overall, there were 202 recorded visits to the metabolic clinic during the pre-defined diagnostic period (the first two months after birth, as almost all cases were ascertained through newborn screening). This represented an average of 1.7 visits (95% confidence interval, 1.5–1.9 visits) per child during which they underwent biochemical and molecular genetic testing to establish a diagnosis of MCAD deficiency.

**Fig. 1:**
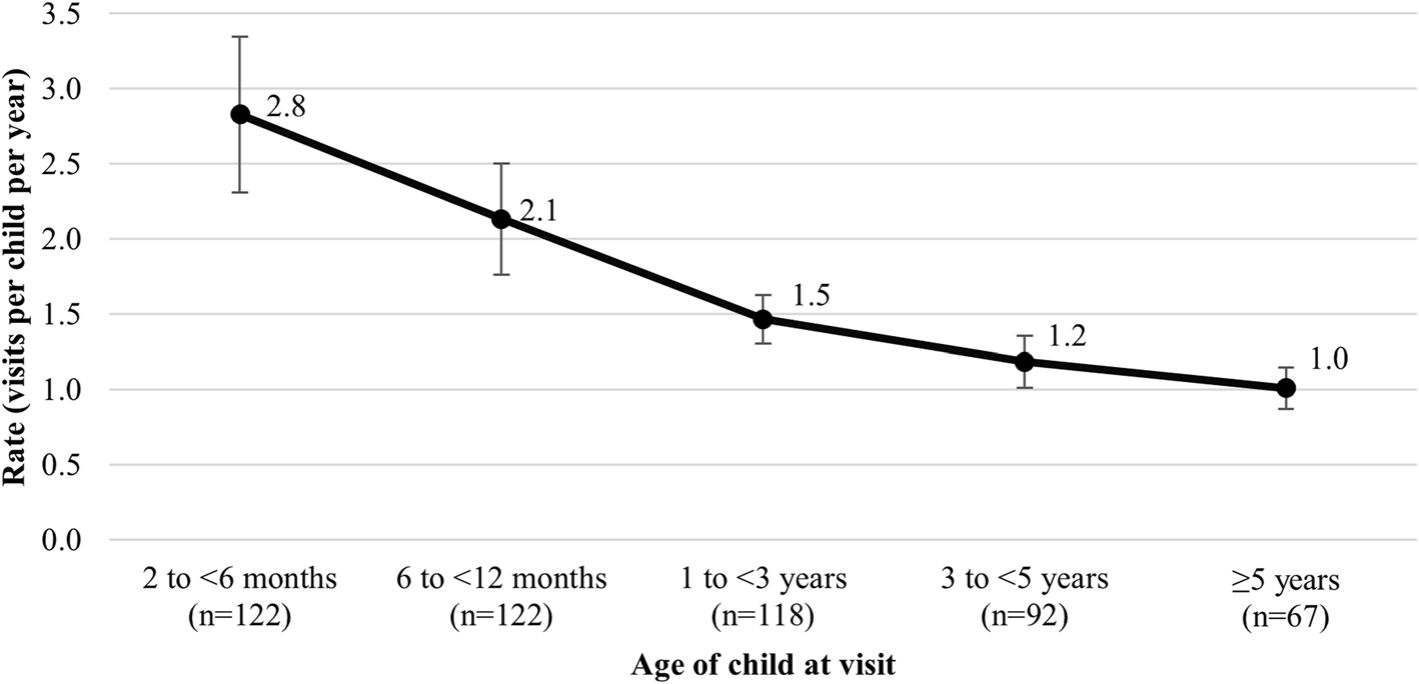
Follow-up visits per child per year to the metabolic clinic, by age at the visit. Error bars represent 95% confidence intervals using the normal approximation to the Poisson distribution. One child may contribute to multiple age groups due to longitudinal follow-up

There was a total of 945 follow-up visits to the metabolic clinic among eligible children at 2 months of age and older. The frequency of visits to the metabolic clinic over time was highest from 2–6 months of age (2.8 visits per child per year), with a gradual, but sustained decline thereafter (2.1 visits per child per year from 6–12 months, 1.5 visits per child per year from 1–3 years, 1.2 visits per child per year from 3–5 years, and 1.0 visits per child per year at 5 years of age and older) (Fig. 1). Among centres treating five or more children with MCAD deficiency, the centre-specific trends in visit rates by child age were mostly consistent with the overall trend (see Additional file 1). There was, however, considerable variation in the magnitude of the rates, likely due to random variation, but possibly reflecting differences in clinic- and clinician-specific management practices.

### Data quality: emergency department visits

Emergency department visits were measured at each metabolic clinic visit, based on the number of trips to the emergency department that had occurred since the most recent previous clinic visit and the reasons prompting those visits. This information was generated from records in the child’s metabolic chart, either derived from clinician report based on the interaction with the family during the clinic visit or from emergency department records embedded in the metabolic chart.

#### Completeness

The number of times that the child had visited the emergency department since their last clinic visit was recorded for approximately 95% of metabolic clinic visits (Table 2) but this varied widely among centres and among individual participants (data not shown). Among 389 recorded emergency department visits for the 124 participants, 94 visits (24%) were missing the exact calendar date of the visit and five visits (1%) were missing the reason for which the child had received emergency care (the latter included unintelligible entries).

**Table 2:** Highlighted findings for core outcomes according to each data quality concept

Emergency department use	Completeness: Presence or absence and number of emergency department visits since the last clinic visit was reported at 95% of clinic visits Conformance: Exact dates of emergency department visits were not always reported, but the age at the visit could often be inferred if it occurred between two clinic visits at known ages Plausibility: Although exhibiting similar trends by age, observed rates are underestimated when compared to a previously published study in Ontario using health care administrative data. Only 42% of expected visits were recorded in the chart
Fasting times	Completeness: Recommended fasting times were updated during approximately 39% of visits. Clinic-specific fasting protocols were provided by some centres, leading to a lack of patient-specific reporting in the chart Conformance: Fasting prescriptions were frequently reported as a range of time, based on the presence/absence of other interventions, or based on specific times of day Plausibility: Median fasting times that were explicitly recorded in this sample followed published recommendations by age
Metabolic decompensation	Completeness: Results of monitoring tests that were expected to be ordered were frequently missing and variation was noted in the level of detail recorded or abstracted Conformance: Episodes were ascertained mainly based on their associated manifestations and rarely directly reported Plausibility: Median age at decompensation roughly followed known ages during which children with MCAD deficiency commonly exhibit symptoms
Death	Fortunately, death occurred extremely rarely in this cohort. Therefore, data quality for this outcome was not able to be evaluated

#### Conformance

The majority of emergency department visits were recorded using validated date fields with accompanying text fields for the reason prompting the visit. However, among the 94 visits without an exact date, 22 emergency department visits had a partial time period entered in a separate open-ended comment (e.g., the month or season within a particular year). For 59 visits (63% of all visits with missing information for calendar date), it was possible to infer at least the approximate age of the child at the time of the visit since it occurred temporally between two clinic visits at known ages.

#### Plausibility

There were no duplicates for data values identifying unique emergency department visits and all emergency department visits were reported as occurring after the participant’s date of birth. Approximately 25 children had no recorded emergency visits during their follow-up over an average period of 4.6 years. Based on a previous study in a sample of children with MCAD deficiency in Ontario, Canada, [20] approximately 920 emergency department visits were expected in the present study cohort. However, among 124 eligible participants, there were only 389 emergency department visits recorded, representing 42% of expected visits.

**Fig. 2:**
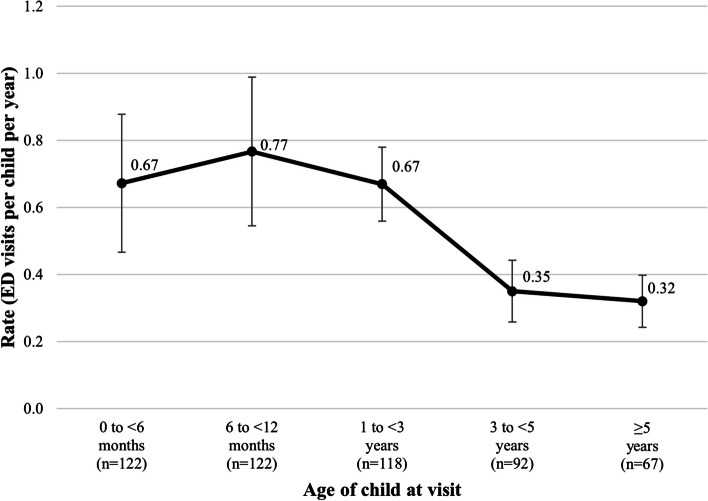
Emergency department visits per child per year, by age at the visit. Error bars represent 95% confidence intervals using the normal approximation to the Poisson distribution. One child may contribute to multiple age groups due to longitudinal follow-up

Rates of visits to the emergency department (Fig. 2) were lower than expected within all age groups based on comparison to the previously published Ontario study [20]. For example, on average, children with MCAD deficiency in the Ontario study visited the emergency department at a rate of 1.5 visits per child per year within the first 6 months of age and 2.5 visits per child per year between 6 months and 1 year of age. For children in the present study cohort, however, these rates were 0.67 and 0.77 metabolic chart-recorded emergency department visits per child per year on average during the same time periods, respectively. The general trend across age groups was similar across the two studies, and both documented the highest observed frequency of emergency department use as occurring between 6 and 12 months of age. The most common reasons for seeking emergency care were similar between our cohort and the previous Ontario study as well: nausea/vomiting (131 visits), upper respiratory tract infections with or without cough (86 visits), and fever (66 visits). Conversely, the frequency of inpatient hospitalizations over time aligned more closely with the published health care administrative data (see Additional file 1), [20] with 68% (190/280) of expected hospital admissions captured in chart-abstracted data.

### Data quality: fasting

Fasting refers to a prescribed maximum period without food or drink as tolerated when well (under usual circumstances) or during intercurrent illness (to prevent acute manifestations of MCAD deficiency). An increased fasting tolerance may be reflective of, or change in response to, improved clinical status of the patient with respect to MCAD deficiency [26].

#### Completeness

Eighty-one children (65%) were prescribed an initial treatment pertaining to fasting avoidance with 67 prescriptions outlining specific details regarding the maximum number of hours recommended without feeding when well. Beyond the initial treatment, four centres (treating 18 children) did not record prescribed maximum fasting times and an additional eight children from other centres were missing fasting values at every metabolic clinic visit. There was an update to the prescribed diet (e.g., energy, fat, carbohydrates) and composition (e.g., supplements) during approximately 52% of the metabolic clinic visits. It was unknown whether any update occurred during 24 (2%) of the metabolic clinic visits. For the metabolic clinic visits during which there was an update to the overall diet, the prescribed fasting time was recorded approximately 74% of the time; thus fasting time was recorded during 39% of all metabolic clinic visits (Table 2). When fasting time was not recorded, it may have been missing, unknown, or unchanged from the previous visit.

#### Conformance

When an acceptable fasting range was provided instead of a specific amount of time (e.g., a prescribed maximum fasting range of 8 to 10 h), we considered the median value of the range to define the fasting time. Some children were prescribed different fasting times for daytime versus overnight; thus, we defined the child’s fasting time as the larger value (maximum amount of time). If a child’s fasting time was defined based on the presence of an additional source of carbohydrates (e.g., fasting with or without cornstarch), we defined the fasting time as the value without extraneous intervention (the lower value) since this represented the amount of time tolerated in normal physiological states.

#### Plausibility

For a number of children, there were no updates to the prescribed diet for long periods of time. As a result, if a lack of update in a metabolic chart were to be considered equivalent to an unchanged prescribed maximum tolerated fasting time (i.e., if we were to carry forward the last explicitly recorded fasting time), the recommended duration of fasting would appear much lower than expected at older ages.

When considering only explicitly recorded values, the median prescribed fasting time when well was 3.5 h (IQR = 3.3–4.0 h) for children under 6 months of age, 6.0 h (IQR = 5.0–8.0 h) for those aged 6 months up to 12 months, 10.0 h (IQR = 8.5–12.0 h) for those aged 1 year up to 2 years, and 12.0 h (IQR = 10.0–12.0 h) for those aged 2 years and older (Table 3). These lengths of time were in approximate agreement with published recommendations for maximum fasting times for children with MCAD deficiency, [26] with a lower median time identified from 6–12 months of age, corresponding to the age period with the highest rate of emergency department use in this cohort. There was no evidence to suggest that the frequency of fasting prescriptions changed over calendar time within age categories (data not shown).

**Table 3:** Prescribed fasting times in the CIMDRN cohort and in published recommendations

**Age Group**	**Number of patients with at least one recorded prescribed fasting time**^a^	**Prescribed fasting time in the CIMDRN cohort** Median (IQR)	**Published fasting times recommendations** [26]
< 6 months	99	3.5 h (3.3–4.0)	N/A
6 to < 12 months	67	6.0 h (5.0–8.0)	8.0 h
1 to < 2 years	59	10.0 h (8.5–12.0)	10.0 h
≥ 2 years	55	12.0 h (10.0–12.0)	12.0 h

^a^Median values were calculated for children with more than one fasting prescription reported within a single age group, and only for those prescriptions with exact values provided (for example, excluding prescriptions stating only “avoidance of fasting” or “frequent feedings”)

### Data quality: metabolic decompensation

The core outcome set defined metabolic decompensation as an acute episode characterized by one or both of the following cardinal features: 1) hypoglycemia and 2) encephalopathy (e.g., lethargy, seizure). Additional supportive evidence based on the results of biochemical tests could include at least one documented abnormal level of substances in the blood indicative of an altered physiological state (e.g., depleted blood glucose, elevated liver aminotransferases) [19].

#### Completeness

The components required to ascertain a decompensation were routinely available within the medical chart (e.g., the results of biochemical tests, reasons for seeking emergency care, acute manifestations). However, identifying an event required clinical judgement influenced by a variety of factors and circumstances, which rendered its derivation complicated. Temporal association of laboratory findings or biomarkers with diagnoses or symptoms was not always possible based on inconsistency in recording of exact dates. In addition to the lack of completeness in reporting of emergency department visits and hospital admissions, the ability to identify metabolic decompensations from abstracted data was limited when no monitoring tests were recorded during these visits and admissions, and when the reasons prompting care were non-specific (Table 2). To this end, there existed large variation among treatment centres in the level of detail abstracted and/or available in the metabolic chart.

#### Conformance

Research staff extracting data for the cohort study rarely used the data collection forms to specify a metabolic decompensation as a direct disease-specific outcome even when it was otherwise apparent that an episode had occurred based on other reported clinical measures. Thus, we relied on associated complications noted in the data extracted from the metabolic chart based on the language used by the health care team to describe a metabolic decompensation. Furthermore, acute manifestations indicative of a metabolic decompensation were not always associated with a specific date; thus, multiple manifestations occurring over a short period of time could not reliably be considered as mutually exclusive events. On a case-by-case basis and to avoid duplicating events, we considered multiple data items possibly contributing to a metabolic decompensation to be related to a single episode if they were reported within the same general time period (e.g., during a single hospital admission) and could not be reliably separated.

#### Plausibility

We identified 99 unique events among eligible participants comprised of individual clinical indicators (e.g., reasons for emergency department visits and hospital admission, acute/chronic diagnoses and manifestations, and biochemical measurements) potentially contributing to a single metabolic decompensation. This included one of or any combination of the following: i) direct reference to metabolic decompensation (reported during 13 events); ii) laboratory-confirmed blood (< 3.5 mmol/L) or clinician-reported hypoglycemia (reported during 34 events); or iii) emergency department visits, hospital admissions, or findings reported outside of routine metabolic clinic visits for indications of preventative IV glucose use and/or monitoring for hypoglycemia (reported during 32 events), elevated aspartate and alanine aminotransferases (reported during 18 events), lethargy (reported during 14 events), seizures (reported during nine events), or direct reference to possible encephalopathy (reported during two events).

Two raters (PC, MTG) independently assessed abstracted data items for the 99 potential events. Thirty-five events were resolved as episodes of metabolic decompensation by both raters. An additional 20 events were identified as metabolic decompensations by one of two raters only; 19 of these were classified as episodes of decompensation by rater 1 and not by rater 2. The remaining events were not considered decompensations by either rater. Inter-rater agreement yielded a Cohen’s kappa statistic of 0.61 (95% confidence interval, 0.45–0.76), indicating moderate to substantial agreement [27]. The lack of very high interrater disagreement was explained by differing interpretation of the requirement for hypoglycemia. Unless there was documented hypoglycemia warranting intervention, raters agreed that isolated reports of intravenous glucose administration (or possible evidence thereof, such as elevated blood glucose levels during hospital admissions) were likely to be precautionary measures taken to avoid fatty acid oxidation and insufficient to confirm a metabolic decompensation. However, we also recognized the difficulty in excluding a metabolic decompensation event when there was no available chart-abstracted glucose level prior to intravenous administration of glucose. In all cases agreed upon by both raters, there was a direct report of a metabolic decompensation and/or evidence for hypoglycemia, therefore we considered these to be “definite” metabolic decompensation events. In 19 of the 20 remaining cases, a glucose level prior to intravenous administration was unavailable, and we considered these “possible” metabolic decompensation events (Table 4).

**Table 4:** Age based rates of metabolic decompensation, estimated by one or two raters identifying a decompensation

**Age Group**	**Number of potential events considered**	**No. events labelled as metabolic decompensations**	**Rate per 100 children per year (95% CI)**^a^
**Overall**			
Definite	99	35	5.0 (3.6–6.9)
Definite + Possible		55	7.9 (6.0–10.2)
** < 12 months**			
Definite		16	13.0 (7.8–21.0)
Definite + Possible	38	22	18.0 (11.7–27.1)
** ≥ 12 months**			
Definite		19	3.3 (2.1–5.1)
Definite + Possible	61	33	5.7 (4.0–8.0)

a) A single child may contribute to both age groups due to longitudinal follow-up

b) “Definite” = Chart-documented hypoglycemia or explicit documentation of metabolic decompensation, “Possible” = cases without chart-documented hypoglycemia prior to intravenous glucose administration

^a^95% confidence intervals calculated using the exact Poisson distribution

Prior to 12 months of age, on average, there were approximately 13–18 episodes of metabolic decompensation per 100 children per year depending on the number of raters who endorsed that one had occurred (Table 4). Among children 12 months of age or older, on average, there were approximately 3 to 6 episodes of metabolic decompensation per 100 children per year. Overall, the median age of metabolic decompensation was approximately 12 to 15 months among episodes that could be ascribed an exact age (i.e., with exact dates available) [28, 29]. Metabolic decompensations are most common between 6 and 18 months of age [30]. Similar to our results, in a previous study, among a sample of 114 children with MCAD deficiency who had previously experienced an acute crisis, the median age at first manifestation was approximately 18 months [31].

### Data quality: death

Since children in this cohort were almost exclusively ascertained by newborn screening, as expected from early identification and initiation of treatment, [1] the core outcome of death was fortunately rare (n < 5), occurring only in few neonates exhibiting highly adverse clinical features prior to diagnosis. Therefore, the data quality related to this outcome could not be evaluated. We note that children who died in the early neonatal period without a confirmed diagnosis of MCAD deficiency prior to death (i.e., those who died prior to a positive newborn screening result and subsequent referral) would not have been eligible to participate in the CIMDRN cohort study and thus would not have been identified as part of the present research; the cohort study enrolled only those children who had a confirmed MCAD deficiency diagnosis and received care at a participating clinic and thus we were only able to identify mortality occurring during the study period.

## Discussion

This study sought to evaluate the quality of existing metabolic chart data and its future suitability for measuring select core outcomes from a COS for MCAD deficiency: emergency department use, fasting time, metabolic decompensation, and death. We found a decreasing rate of follow-up visits to the metabolic clinic by age, consistent with evidence suggesting that the highest risk periods for developing complications occur in younger age groups [31]. Based on the frequency of follow-up visits to the metabolic clinic, an opportunity exists to measure these clinical core outcomes in the metabolic clinic setting at least once per year regardless of patient age. Although other COS studies have defined specific time points for measurement of core outcomes according to disease-specific milestones and timelines, [32] there is no published benchmark for the frequency of collection of core outcomes in children. For MCAD deficiency, the minimum frequency of visits required to capture meaningful change in an outcome may depend on the outcome itself, its measurement scale’s responsiveness to change, and, in studies evaluating management strategies, the specific treatment being evaluated as it relates to the expected length of time until a change in outcome can be expected. Therefore, alternative strategies to collect clinical core outcomes from children who visit the metabolic clinic less frequently may be required; improvements to the provision of virtual ambulatory health care and to shared electronic health records during the SARS-COv2 pandemic may present opportunities for such alternatives.

We believe that rates of emergency department visits were likely to have been underestimated when relying on metabolic chart data alone, but otherwise followed anticipated trends by age. We found the highest rate of emergency department visits between 6 and 12 months of age, which is consistent with previous literature and corresponds with the highest risk period for experiencing metabolic decompensations [33]. It is well known that the accuracy of medical records data depend on the type of data collected; demographic, outcome, and discharge information collected as part of standardized sections of the chart have been found to have the highest accuracy [34]. Data quality may be further maximized when relying on outcomes, such as emergency department visits, that rely in large part on a binary response, such as the presence or absence of a health event. However, *post-hoc* follow-up investigation with research staff at each participating centre suggested that information captured within the metabolic chart pertaining to emergency department visits often corresponded to visits that had occurred at same health care institution’s emergency department. Emergency department visits occurring at other centres, such as at local community hospitals, may not have been captured unless the information had been parent-reported, forwarded by the emergency care team, or otherwise requested by the metabolic team. There was an improved comparability of rates of inpatient hospitalizations with gold standard measures relative to rates of emergency department visits [20]. This suggests that emergency department visits warranting more critical and longer-term care may be more rigorously documented in the metabolic chart. Collectively then, when relying on metabolic chart data, we hypothesize that whether an emergency department visit that occurs is captured depends on the nature and severity of the visit and its relevance to a child’s disease as well as whether the visit occurred at the same institution (which in turn seems likely to vary according to both disease severity and geographic proximity of the child’s residence to the hospital with which the outpatient metabolic clinic is associated). The fact that emergency visits occurring at community hospitals may be missed from metabolic charts and that these visits may be distinct in nature from those that are captured suggests a need for additional pro-active measures to systematically capture emergency care. Strategies may include supplementing metabolic chart data with patient- or parent-reported forms and linkable comprehensive health care administrative data.

Measuring prescribed maximum fasting times from metabolic chart data that had been documented for clinical rather than research purposes was problematic. Although similar to emergency department visits in that the quality of reporting of this outcome relied on the presence or absence of a fasting prescription recorded in the metabolic chart, [34] it is possible that updates were often the product of informal discussions between a clinician and a patient or their family member/caregiver that are not documented. We found that some centres provided families/caregivers with a clinic-specific protocol describing standard recommended fasting times according to age periods. In such cases, only exceptions to this algorithm may have been recorded in the metabolic chart. Consequently, recommended fasting times were updated highly infrequently in patients’ charts, particularly at certain centres. Therefore, consistent reporting of fasting times for prospective research will require the use of clinic report forms completed at each metabolic clinic visit that appropriately capture prescribed diets and recommended fasting times.

In this study, the agreement among metabolic physicians was considered moderate to substantial in terms of characterizing abstracted data items as episodes of metabolic decompensation. The nature of the disagreements in classification between the two observers suggested that each had a differential threshold for an event to be considered a metabolic decompensation. For example, hypoglycemia was a common clinical characteristic among events identified as decompensations by both raters, but invariably reflects a late symptom of metabolic decompensation [33]. A factor rendering it difficult to measure occurrence of metabolic decompensations consistently is that emergency care for MCAD deficiency is often focused on mitigating risk and thus it can be difficult to distinguish a “near miss” (i.e., decompensation avoided due to an intervention) from a full blown decompensation. It was also difficult to temporally associate clinical manifestations contributing to a single event based on metabolic chart data. Certain features occurring alone may not always constitute a true decompensation and may require clinical expertise and judgement (e.g., based on the age of child, the perceived severity of disease, temporal association with other indicators, “susceptibility” from prior events, and other comorbidities). In general, diagnostic information has been found to have lower accuracy compared to other data, which can introduce systematic errors in the absence of standardized definitions and non-adherence to those definitions [34]. Thus, this outcome requires a clear operational definition meaningful to a large number of clinicians treating children with MCAD deficiency. The refinement and application of practice guidelines to include the diagnosis of metabolic decompensation events and for acute management of patients with MCAD Deficiency could also both benefit patient care and also the usability of chart abstracted data for research and quality improvement.

Metabolic centres with both paper-based charts and electronic health records participated in the initial cohort study, with some having referenced both formats of the chart concurrently and others having transitioned to their electronic system during the latter period of the clinic visit eligibility. It was not possible to formally examine whether the quality of metabolic chart data differed based on the format of the chart. Anecdotally, however, we anticipate that differences between centres in the quality of data and reporting are not dependent on the format of the chart due to the inconsistent way that clinical data are currently captured even in electronic forms. Standardization of clinical forms and field definitions (whether paper or electronic) across clinics to the extent possible will likely be required.

The data quality issues we identified currently limit our ability to leverage real-world clinical data for children with MCAD deficiency in research and quality assurance initiatives to inform and improve care. These findings indicate a need for investment in platforms and infrastructure to support high quality routine outcome monitoring in clinical settings to produce usable data that can be synthesized and compared across care centres. Such platforms will facilitate evidence generation to ultimately improve care and outcomes for children with MCAD deficiency, for example through high-quality prospective multi-centre registries that can provide long-term natural history data and support pragmatic trials.

### Limitations

We capitalized on the availability of existing cohort data abstracted from metabolic charts to evaluate reporting of core outcomes for pediatric MCAD deficiency. However, because the CIMDRN cohort study was not specifically designed to collect our core outcome set, it is possible that we underestimated the extent to which the information required to document core outcomes was present in patients’ metabolic charts compared with the variables that we could extract from the database. Specifically, a health event or variable that was indicative of a particular outcome may be inadequate or unavailable in the longitudinal database simply because the definitions and measurement criteria for that outcome were selected a posteriori relative to the cohort data collection. A further limitation to our study is the current scarcity of data available for older children. Included data on older age groups were populated mainly by children with longer follow-up and born in earlier years by nature of the data collection period relative to the years of birth included in this study. Finally, clinicians relied solely on abstracted data items to evaluate the incidence of metabolic decompensations and did not have access to the child’s medical history, which may have influenced their clinical judgement and their ability to correctly identify decompensation episodes.

## Conclusions

Opportunities to record core outcomes at the metabolic clinic occur at least annually for children with MCAD deficiency. Methods to comprehensively capture emergency care received at outside institutions are needed. To reduce substantial heterogeneous recording of core outcome across treatment centres, improved reporting standards are required for consistent documentation of recommended fasting times and a consensus definition for metabolic decompensations needs to be developed and implemented.

### Supplementary Information

**Additional file 1: Supplemental Figure 1.** Rates of follow-up visits to the metabolic clinic stratified by treatment centre (A-H). **Supplemental Figure 2.** Rates of inpatient hospitalizations according to the age at the visit.

## Data Availability

The datasets generated and/or analysed during the present study are not publicly available in order to protect the privacy of the study participants. Other materials used for this study (e.g., data collection tools, data dictionary, detailed data privacy/protection procedures, etc.) are available from the corresponding author (Pranesh Chakraborty, Children’s Hospital of Eastern Ontario, pchakraborty@cheo.on.ca, 1 (613) 737–7600 extension 3437) upon reasonable request.

## Abbreviations

COMET: Core Outcome Measures in Effectiveness Trials
CIMDRN: Canadian Inherited Metabolic Diseases Research Network
IMD: Inherited metabolic diseases
MCAD: Medium-chain acyl-CoA dehydrogenase
REDCap: Research Electronic Data Capture
