# Supplementary material for: Assessing the quality and value of metabolic chart data for capturing core outcomes for pediatric medium-chain acyl-CoA dehydrogenase (MCAD) deficiency
Source: BMC Pediatr. 2024 Jan 13;24:37. doi: 10.1186/s12887-023-04393-4 (PMC10787451; doi:10.1186/s12887-023-04393-4)
Supplement: Supplementary file 1 — Additional file 1: Supplemental Figure 1. Rates of follow-up visits to the metabolic clinic stratified by treatment centre (A-H). Supplemental Figure 2. Rates of inpatient hospitalizations according to the age at the visit. [file 12887_2023_4393_MOESM1_ESM.docx]

**Supplemental Figure 1.** Rates of follow-up visits to the metabolic clinic stratified by treatment centre (A-H).

***** One child may contribute to multiple age groups due to longitudinal follow-up

* Only includes centres treating 5 or more children

**Supplemental Figure 2.** Rates of inpatient hospitalizations according to the age at the visit.

* Error bars representing 95% confidence intervals using the normal approximation to the Poisson distribution

* One child may contribute to multiple age groups due to longitudinal follow-up
